# Clinical Utility of the Paed‐EQUAL Candida Score in Paediatric Candidemia: A Real‐World Cohort Study

**DOI:** 10.1111/myc.70207

**Published:** 2026-07-10

**Authors:** Kubra Aykac, Ilgın Sirin, Turgut Ozdemir, Osman Oguz Demir, Aylin Irmak Kuruc, Hanife Avcı, Ali Bulent Cengiz, Yasemin Ozsurekci

**Affiliations:** ^1^ Department of Pediatric Infectious Diseases Hacettepe University Faculty of Medicine Ankara Türkiye; ^2^ Department of Pediatric Diseases Ankara, Turkey, Hacettepe University Faculty of Medicine Ankara Türkiye; ^3^ Department of Biostatistics Hacettepe University Faculty of Medicine Ankara Türkiye

**Keywords:** candidemia, children, Paed‐EQUAL Candida score

## Abstract

**Background:**

The Paed‐EQUAL Candida Score was developed to assess adherence to guideline‐recommended management in paediatric candidemia; however, its clinical utility in predicting outcomes remains unclear, particularly in paediatric populations where validation data are scarce.

**Methods:**

We conducted a retrospective observational study including paediatric patients diagnosed with candidemia at a tertiary‐care centre between 2014 and 2024. The Paed‐EQUAL Candida Score was calculated for each patient, and its association with 30‐day and 90‐day mortality was evaluated using ROC curve analysis, and optimal cut‐off values were explored.

**Results:**

A total of 127 paediatric patients with candidemia were included, of whom 17 (13.4%) died within 30 days and 27 (21.3%) within 90 days. Central venous catheter removal was significantly associated with improved survival at both 30 days (76.4% vs. 35.3%, *p* = 0.001) and 90 days (77% vs. 48.1%, *p* = 0.007). Higher adherence to Paed‐EQUAL Candida Score components was observed among survivors, with significantly higher median scores in survivors compared to non‐survivors at 30 days (17 [IQR 14–18] vs. 14 [12–16], *p* = 0.026). However, the score indicated modest discriminative ability for predicting mortality, with an AUC of 0.667 (95% CI 0.577–0.748) for 30‐day mortality and no significant predictive value for 90‐day mortality (AUC 0.594, 95% CI 0.503–0.680).

**Conclusion:**

The Paed‐EQUAL Candida Score demonstrated limited prognostic performance in paediatric candidemia and may have constraints in its ability to support clinical decision‐making, particularly given its modest discriminative capacity. While it remains a potentially useful tool for assessing adherence to quality‐of‐care measures, further refinement or development of paediatric‐specific and outcome‐oriented scoring systems may be needed to improve risk stratification and prediction of clinical outcomes.

## 1 Introduction

Childhood candidemia remains a serious clinical entity associated with substantial morbidity and mortality, with reported mortality rates ranging from 15% to 30% in paediatric populations [1, 2, 3, 4]. The burden of disease is particularly pronounced among children with underlying comorbidities and exposure to healthcare‐associated risk factors, including central venous catheters, broad‐spectrum antibiotic therapy, and intensive care unit admission. Importantly, previous studies, including data from our centre, have demonstrated that timely management interventions, particularly early catheter removal, are strongly associated with improved survival [5, 6, 7]. Despite the availability of international guidelines for the management of candidemia [6], adherence to recommended diagnostic and therapeutic interventions remains variable in real‐world paediatric practice [8]. In addition, most recommendations are largely derived from adult data, and objective tools specifically designed to assess the quality of care in paediatric candidemia are limited [1, 9, 10]. To address this gap, the Paed‐EQUAL Candida Score was recently developed to quantify adherence to guideline‐recommended care in children with candidemia, incorporating key diagnostic, therapeutic, and follow‐up components into a structured and measurable framework [8]. While similar adherence‐based scoring systems, such as the EQUAL Candida Score, have been evaluated in adult populations with respect to clinical outcomes, their applicability and prognostic relevance in paediatric cohorts remain insufficiently explored [11, 12]. Therefore, given the potential discrepancies between guideline recommendations and real‐world clinical practice in children, external validation of the Paed‐EQUAL Candida Score in independent paediatric cohorts is warranted. In this study, we aimed to evaluate both the clinical utility and prognostic performance of the Paed‐EQUAL Candida Score in a real‐world cohort of paediatric patients with candidemia, with a particular focus on its association with mortality outcomes.

## 2 Method

### 2.1 Study Design and Patient Selection

We conducted a retrospective, single‐centre observational study including all consecutive paediatric patients (< 18 years) diagnosed with candidemia between January 2014 and December 2024. Demographic, clinical, microbiological, and management‐related data were extracted from electronic medical records. The Paed‐EQUAL Candida Score was calculated for each patient according to the published European Confederation of Medical Mycology (ECMM) paediatric scoring system, which evaluates adherence to guideline‐recommended diagnostic, therapeutic, and follow‐up measures [8]. Due to the limited number of neonates and patients without central venous catheters, subgroup‐specific analyses were not feasible. Therefore, the scoring system was applied only to post‐neonatal paediatric patients with central venous catheters. All score components were retrospectively assessed based on documented clinical practice.

Data collected included Candida species/species complex (SC) identification, underlying risk factors, treatment characteristics, and clinical outcomes. Isolates were identified via conventional methods (including colony morphology, germ tube test, thermotolerance test, and urease activity) and assimilation profiles as assessed by ID32C (BioMérieux, France) between 2014 and 2017 and by MALDI‐TOF MS (Bruker, Germany) between 2018 and 2024. The study was approved by the Hacettepe University Non‐Interventional Ethical Committee (GO 26/327).

### 2.2 Definitions

Invasive candidiasis was defined according to international guidelines as bloodstream infection due to Candida species in the presence of compatible clinical features, requiring both microbiological confirmation and supportive clinical evidence [6]. Patients were classified as survivors or non‐survivors based on all‐cause mortality occurring within 30 and 90 days after the first positive blood culture [10, 12]. Thirty‐day and 90‐day mortality were selected to facilitate comparison with previous studies evaluating the prognostic performance of the EQUAL Candida Score and related candidemia outcome studies.

### 2.3 Statistical Analysis

The analyses were conducted using the free and open‐source software R (version 4.5.2, https://cran.r‐project.org) and the SPSS for Windows Version 23.0 statistical package (Chicago, IL), with the assistance of an academic biostatistician. Normality of the data was assessed using the Kolmogorov–Smirnov test, and variance homogeneity was tested using Levene's test. Descriptive statistics were presented as median (25th percentile–75th percentile) and frequencies (percentages) as appropriate. To compare the differences between the groups, the Mann–Whitney U test was used for continuous variables, and the Yates continuity correction Chi‐square test, Fisher's Exact test, and Fisher–Freeman–Halton test, as appropriate, were used for categorical variables. Variables with *p* < 0.20 as a result of univariate analysis were determined as mortality variables and included in multiple binary logistic regression models. Odds ratios with 95% confidence intervals were calculated. Model calibration, specifically goodness‐of‐fit, was evaluated using the Hosmer–Lemeshow test, with *p* > 0.05 indicating a robust model that cannot be rejected. Model fit was evaluated using the classification table. ROC curve analysis was conducted to assess the ability of the Paed‐EQUAL Candida Score to discriminate 30‐day and 90‐day mortality, and the optimal cut‐off points were identified based on the Youden index. The “pROC” package was used to calculate the area under the curve (AUC) [13]. A *p*‐value of less than 5% was considered statistically significant.

## 3 Results

### 3.1 Patient Characteristics and Mortality Outcomes

A total of 127 paediatric patients with candidemia were included, of whom 17 (13.4%) died within 30 days and 27 (21.3%) within 90 days. The median age was 21 months (IQR 6–61), and 59.1% were male, with no significant differences between survivors and non‐survivors at either time point (Table 1).

**TABLE 1:** Clinical characteristics, comorbidities and outcomes of patients with candidemia, stratified by 30‐day mortality.

	All patients (*n* = 127)	Survivors (*n* = 110)	Non‐survivors (*n* = 17)	*p*
Age (month), median, IQR	21 (6–61)	22 (7–63)	7 (3–40)	0.088^a^
Male, *n* (%)	75 (59.1%)	65 (59.1%)	10 (58.8%)	> 0.05^b^
Underlying diseases				0.085^c^
Haematologic malignancy	6 (4.7%)	6 (5.5%)	—	
Oncologic malignancy	32 (25.2%)	24 (21.8%)	8 (47.1%)	
Primary immunodeficiency	4 (3.1%)	4 (3.6%)	—	
Neurologic	17 (13.4%)	14 (12.7%)	3 (17.6%)	
Metabolic	9 (7.1%)	9 (8.2%)	—	
Gastrointestinal	23 (18.1%)	23 (20.9%)	—	
Cardiac	17 (13.4%)	12 (10.9%)	5 (29.4%)	
Renal	6 (4.7%)	6 (5.5%)	—	
Others	10 (7.9%)	9 (8.2%)	1 (5.9%)	
HSCT/SOT	3 (2.4%)	3 (2.7%)	—	
Candida spp.				0.154^c^
*C. albicans*	45 (35.4%)	37 (33.6%)	8 (47.1%)	
Non*‐albicans Candida*	75 (59.1%)	68 (61.8%)	7 (41.2%)	
Multiple *Candida* spp.	7 (5.5%)	5 (4.5%)	2 (11.8%)	
Risk factors, *n* (%)				
Immunocompromised	61 (48%)	52 (47.3%)	9 (52.9%)	0.861^b^
Neutropenia	107 (84.3%)	94 (85.5%)	13 (76.5%)	0.471^d^
Total parenteral nutrition	33 (26%)	28 (25.5%)	5 (29.4%)	0.769^d^
Prematurity	23 (18.1%)	20 (18.2%)	3 (17.6%)	> 0.05^d^
IMV	38 (29.9%)	29 (26.4%)	9 (52.9%)	0.052^b^
PICU	47 (37%)	38 (34.5%)	9 (52.9%)	0.233^b^
Bacterial infection*	63 (49.6%)	55 (50%)	8 (47.1%)	> 0.05^b^
Exposure to antibiotics*	102 (80.3%)	87 (79.1%)	15 (88.2%)	0.522^d^
Catheter removal, *n* (%)				0.001^d^
Yes	90 (70.9%)	84 (76.4%)	6 (35.3%)	
No	37 (29.1%)	26 (23.6%)	11 (64.7%)	
Treatment, *n* (%)				0.154^c^
Fluconazole	13 (10.2%)	11 (10%)	2 (11.8%)	
Caspofungin	94 (74%)	80 (72.7%)	14 (82.4%)	
Amphotericin B	3 (2.4%)	2 (1.8%)	1 (5.9%)	
Combined	17 (13.4%)	17 (15.5%)	—	

Abbreviation: PICU, Paediatric Intensive Care Unit.

* In the last 1 month. IMV: Invasive mechanical ventilation.

^a^ Yates continuity correction chi‐squared test.

^b^ Fisher's exact test.

^c^ Fisher‐Freeman‐Halton test.

^d^ Mann–Whitney U test.

Oncologic malignancies were the most common underlying condition (25.2%), followed by gastrointestinal (18.1%) and neurologic disorders (13.4%). Although haematologic malignancies were more frequent among non‐survivors at 30 days (47.1% vs. 5.5%), the overall distribution of underlying comorbidities did not differ significantly for 30‐day mortality (*p* = 0.085). However, a significant difference emerged at 90 days (*p* = 0.038), driven primarily by a higher proportion of oncologic malignancies among non‐survivors (40.7% vs. 21%).

Non‐albicans Candida species predominated (59.1%), with no differences between groups. Among clinical risk factors, neutropenia (84.3%) and prior antibiotic exposure (80.3%) were highly prevalent across the cohort, with no significant differences between survivors and non‐survivors (*p* = 0.471 and *p* = 0.522, respectively).

Invasive mechanical ventilation was more frequent among non‐survivors (52.9% vs. 26.4% at 30 days; 48.1% vs. 25% at 90 days), reaching statistical significance only for 90‐day mortality (*p* = 0.036).

Catheter removal emerged as one of the most prominent factors associated with survival, being significantly less frequent among non‐survivors at both 30 days (35.3% vs. 76.4%, *p* = 0.001) and 90 days (48.1% vs. 77%, *p* = 0.007). Overall, 26 patients (20.5%) had ports, whereas 101 (79.5%) had temporary or tunnelled central venous catheters. Ports were significantly more common among non‐survivors than survivors at both 30 days (41.2% vs. 17.3%, *p* = 0.046) and 90 days (37.0% vs. 16.0%, *p* = 0.029). Catheter removal was also significantly less frequent in patients with ports than in those with temporary or tunnelled central venous catheters (42.3% vs. 78.2%, *p* = 0.001).

Initial antifungal therapy consisted of caspofungin in 61 patients (48.0%), fluconazole in 40 (31.5%), amphotericin B in 22 (17.3%), and combination therapy in 4 patients (3.1%). At the end of treatment, caspofungin remained the most frequently used antifungal agent (74.0%), followed by combination therapy (13.4%), fluconazole (10.2%), and amphotericin B (2.4%). Among the 40 patients initially treated with fluconazole, only 3 had isolates subsequently found to be resistant to fluconazole. Antifungal treatment strategies did not differ significantly between groups.

### 3.2 Adherence to Paed‐EQUAL Candida Score and Association With Mortality

Adherence to individual Paed‐EQUAL Candida Score components is presented in Table 2. Infectious disease consultation and species identification were achieved in all patients, limiting their ability to discriminate between survivors and non‐survivors. Antifungal susceptibility testing was performed in 81.1% of patients and did not differ between survivors and non‐survivors. Similarly, initial antifungal therapy with an echinocandin or liposomal amphotericin B was appropriately initiated in the majority of patients (89%) with no significant difference between groups.

**TABLE 2:** Adherence to Paed‐EQUAL Candida Score parameters among paediatric patients with candidemia, according to 30‐ and 90‐day mortality.

Quality indicator, *n* (%)		30‐day mortality			90‐day mortality		
	All patients (*n* = 127)	Survivors (*n* = 110)	Non‐survivors (*n* = 17)	*p*	Survivors (*n* = 100)	Non‐survivors (*n* = 27)	*p*
Infectious diseases specialist consultation	127 (100%)	110 (100%)	17 (100%)	—	100 (100%)	27 (100%)	—
Appropriate blood culture volume 6 mL for children 2.1–12.7 kg, 20 mL for children 12.8–36.3 kg	0	0	0	—	0	0	—
Species identification	127 (100%)	110 (100%)	17 (100%)	—	100 (100%)	27 (100%)	—
Antifungal susceptibility testing	103 (81.1%)	89 (80.9%)	14 (82.4%)	> 0.05^a^	82 (82%)	21 (77.8%)	0.826^a^
Echocardiography performed	99 (78%)	88 (80%)	11 (64.7%)	0.206^b^	82 (82%)	17 (63%)	0.063^a^
Ophthalmoscopic examination performed	61 (48%)	58 (52.7%)	3 (17.6%)	0.015^a^	53 (53%)	8 (29.6%)	0.052^a^
Initial antifungal therapy with echinocandin (ECH) or liposomal amphotericin B (L‐AMB)	113 (89%)	98 (89.1%)	15 (88.2%)	> 0.05^b^	88 (88%)	25 (92.6%)	0.733^b^
Treatment initiated ≤ 12 h after communication of positive Candida blood culture result	63 (49.6%)	56 (50.9%)	7 (41.2%)	0.627^a^	48 (48%)	15 (55.6%)	0.631^a^
Delayed treatment initiation > 72 h after communication of a positive Candida blood culture result	35 (27.6%)	30 (27.3%)	5 (29.4%)	> 0.05^b^	30 (30%)	5 (18.5%)	0.346^a^
Antifungal therapy continued for 14 days after the first negative blood culture	100 (78.7%)	93 (54.5%)	7 (41.2%)	< 0.001^b^	83 (83%)	17 (63%)	0.046^a^
CVC removal							0.431^c^
≤ 24 h from diagnosis	5 (3.9%)	4 (3.6%)	1 (5.9%)		3 (3%)	2 (7.4%)	
24–72 h from diagnosis	13 (10.2%)	11 (10%)	2 (11.8%)		10 (10%)	3 (11.1%)	
> 72 h from diagnosis	109 (85.8%)	95 (86.4%)	14 (82.4%)	0.582^c^	87 (87%)	22 (81.5%)	
Follow‐up blood cultures performed on days 3 and 5	90 (70.9%)	84 (76.4%)	6 (35.3%)	0.001^b^	77 (77%)	13 (48.1%)	0.007^a^
Paed‐EQUAL Candida Score, median (Q1–Q3)	16 (13–18)	17 (14–18)	14 (12–16)	0.026^d^	17 (14–18)	14 (12–17)	0.134^d^

Abbreviations: CVC, central venous catheter; EQUAL, European Confederation of Medical Mycology Quality of Clinical Candidemia Management Score.

^a^ Mann‐Whitney U test.

^b^ Yates continuity correction chi‐square test.

^c^ Fisher‐Freeman‐Halton test.

^d^ Fisher's exact test.

However, adherence to several key quality indicators was significantly lower among non‐survivors. Ophthalmoscopic examination was less frequently performed in non‐survivors compared to survivors at 30 days (17.6% vs. 52.7%, *p* = 0.015), with a similar trend observed at 90 days (29.6% vs. 53%, *p* = 0.052).

Markers of follow‐up quality and treatment continuity were also more frequently achieved among survivors. Continuation of antifungal therapy for at least 14 days after the first negative blood culture was significantly more frequent among survivors at both 30 days (54.5% vs. 41.2%, *p* < 0.001) and 90 days (83% vs. 63%, *p* = 0.046). Similarly, follow‐up blood cultures on days 3 and 5 were performed more frequently in survivors at both 30 days (76.4% vs. 35.3%, *p* = 0.001) and 90 days (77% vs. 48.1%, *p* = 0.007).

The median Paed‐EQUAL Candida Score was significantly higher in survivors compared to non‐survivors at 30 days (17 [IQR 14–18] vs. 14 [12, 13, 14, 15, 16], *p* = 0.026), whereas the difference did not reach statistical significance at 90 days (17 [14, 15, 16, 17, 18] vs. 14 [12, 13, 14, 15, 16, 17], *p* = 0.134). These findings suggest that while higher adherence to guideline‐based care is associated with short‐term survival, its ability to predict longer‐term outcomes may be limited.

### 3.3 Multivariable Analysis of Mortality

Multivariable logistic regression analyses for 30‐ and 90‐day mortality are presented in Table 3. In multivariable analysis for 30‐day mortality, continuation of antifungal therapy for at least 14 days after the first negative blood culture was independently associated with survival (OR 5.607, 95% CI 1.659–18.951, *p* = 0.006). Similarly, central venous catheter removal was independently associated with reduced mortality (OR 4.244, 95% CI 1.262–14.270, *p* = 0.019). Follow‐up blood cultures performed on days 3 and 5 were not independently associated with survival in the multivariable model (OR 3.322, 95% CI 0.976–11.307, *p* = 0.055). The model demonstrated excellent calibration (Hosmer–Lemeshow *p* = 0.967) and high overall accuracy (89%).

**TABLE 3:** Multivariable logistic regression analysis for mortality.

30 days mortality			
Risk factors	OR	95% CI	*p*
Constant	0.028	—	< 0.001
Antifungal therapy continued for 14 days after the first negative blood culture	5.607	1.659–18.951	0.006
Follow‐up blood cultures performed on days 3 and 5	3.322	0.976–11.307	0.055
CVC removal	4.244	1.262–14.270	0.019

90 days mortality			
Risk factors	OR	95% CI	*p*
Constant	0.057	—	< 0.001
Follow‐up blood cultures performed on days 3 and 5	3.424	1.261–9.293	0.016
Neutropenia	3.840	1.094–13.484	0.036
Invasive mechanical ventilation	3.806	1.360–10.652	0.011
CVC removal	2.774	1.039–7.404	0.042

*Note:* Hosmer–Lemeshow goodness‐of‐fit test: χ^2^ = 0.560, *p* = 0.967 Overall accuracy: 0.890; Hosmer–Lemeshow goodness‐of‐fit test: χ^2^ = 4.351, *p* = 0.500 Overall accuracy: 0.858.

For 90‐day mortality, follow‐up blood cultures on days 3 and 5 remained independently associated with survival (OR 3.424, 95% CI 1.261–9.293, *p* = 0.016). In addition, neutropenia (OR 3.840, 95% CI 1.094–13.484, *p* = 0.036) and invasive mechanical ventilation (OR 3.806, 95% CI 1.360–10.652, *p* = 0.011) were identified as independent risk factors for mortality. Central venous catheter removal also remained significantly associated with improved survival at 90 days (OR 2.774, 95% CI 1.039–7.404, *p* = 0.042). The model showed acceptable calibration (Hosmer–Lemeshow *p* = 0.500) and good overall accuracy (85.8%).

Overall, these findings suggest that early and sustained adherence to key management components, particularly treatment continuation and source control, plays a central role in short‐term survival, whereas longer‐term outcomes appear to be more strongly influenced by disease severity and ongoing clinical management.

### 3.4 ROC Analysis of Paed‐EQUAL Candida Score

Receiver operating characteristic (ROC) curve analysis demonstrated that the Paed‐EQUAL Candida Score had only modest discriminative ability for predicting 30‐day mortality, with an area under the curve (AUC) of 0.667 (95% CI: 0.577–0.748, *p* = 0.0109). The optimal cut‐off value was ≤ 17, yielding a high sensitivity (94.1%) but low specificity (36.4%), indicating limited ability to accurately distinguish high‐risk patients despite good sensitivity.

For 90‐day mortality, the predictive performance of the score was lower and did not reach statistical significance (AUC: 0.594, 95% CI: 0.503–0.680, *p* = 0.1337). The optimal cut‐off value was ≤ 16, with moderate sensitivity (66.7%) and limited specificity (51.0%).

**FIGURE 1:**
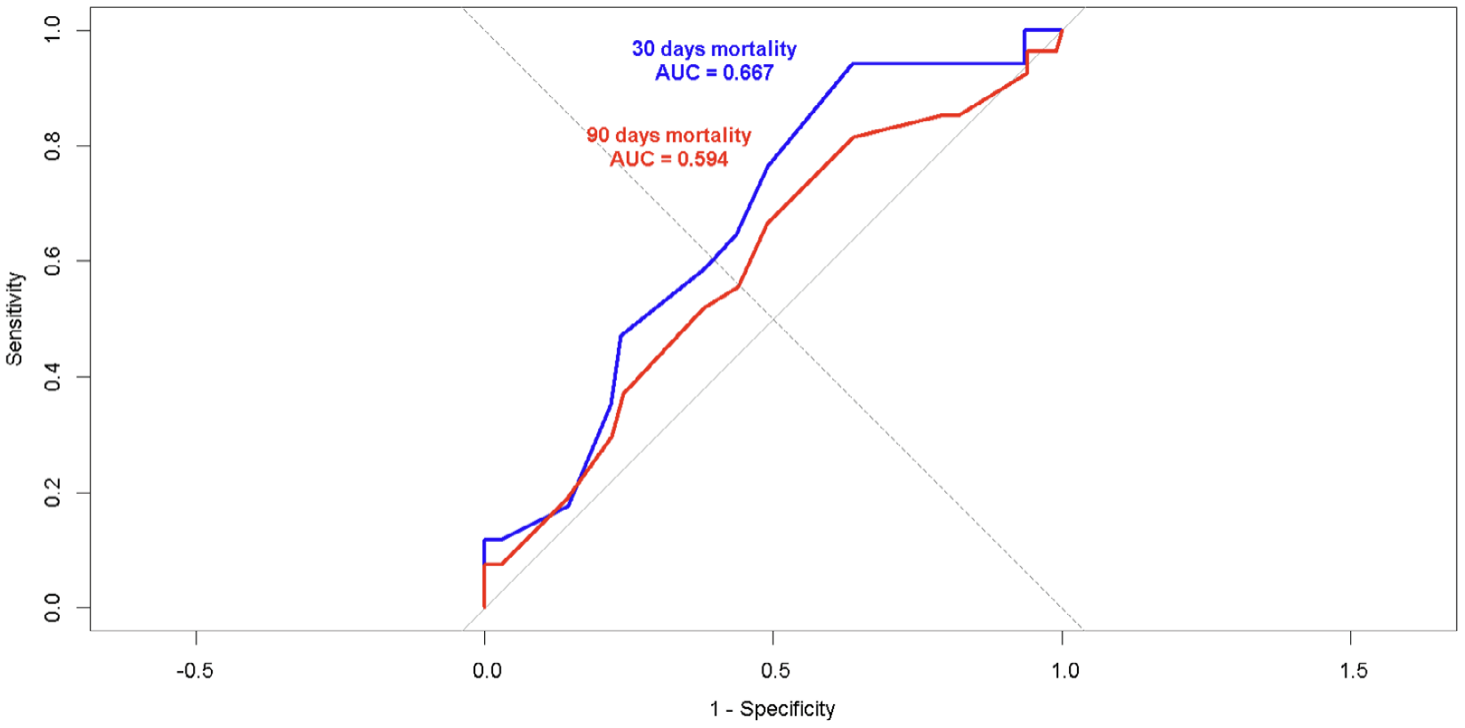
Receiver operating characteristic (ROC) curves of the Paed‐EQUAL Candida Score for predicting 30‐day and 90‐day mortality. The discriminative ability of the score was modest for 30‐day mortality (AUC = 0.667, 95% CI: 0.577–0.748, *p* = 0.0109), with an optimal cut‐off value of ≤ 17 yielding high sensitivity (94.1%) but low specificity (36.4%). For 90‐day mortality, the predictive performance was lower and not statistically significant (AUC = 0.594, 95% CI: 0.503–0.680, *p* = 0.1337), with an optimal cut‐off value of ≤ 16 (sensitivity 66.7%, specificity 51.0%). The diagonal line represents no discriminative ability.

Comparison of the ROC curves for 30‐day and 90‐day mortality suggests that the discriminative performance of the score declines over time, further limiting its utility for longer‐term outcome prediction (Figure 1).

## 4 Discussion

In this study, we externally evaluated the performance of the Paed‐EQUAL Candida Score in a real‐world paediatric candidemia cohort and examined its association with clinical outcomes. We found that while higher adherence to guideline‐recommended management was associated with improved survival, the overall discriminative performance of the score for predicting mortality was limited. Although the score demonstrated high sensitivity for 30‐day mortality, its low specificity substantially reduced its ability to reliably distinguish high‐risk patients. Furthermore, its performance for predicting 90‐day mortality was poor and did not reach statistical significance. Importantly, differences in outcomes appeared to be primarily driven by specific components of care, including particularly treatment continuation, follow‐up blood cultures, and catheter removal, rather than the composite score itself. Overall, these findings indicate that while the Paed‐EQUAL Candida Score may be useful as a sensitive tool for assessing adherence to care, its role is better suited to evaluating quality‐of‐care processes rather than serving as a prognostic tool in clinical decision‐making.

These findings may be explained by inherent limitations of applying adherence‐based scoring systems to paediatric candidemia. Such scores primarily reflect processes of care rather than underlying disease severity, which may limit their prognostic accuracy in paediatric populations.

In paediatric patients, factors such as age‐related immune responses, heterogeneity of underlying conditions, and the central role of source control may have a greater influence on outcomes than adherence to predefined management steps [5]. Moreover, our cohort included patients with diverse underlying diseases and clinical characteristics, which may have influenced the prognostic performance of the Paed‐EQUAL Candida Score. As the score was designed to assess adherence to guideline‐recommended management rather than patient‐related prognostic factors, it does not account for differences in underlying diseases, Candida species, or illness severity, all of which may independently affect mortality outcomes. In adult populations, adherence‐based scoring systems such as the EQUAL Candida Score have consistently demonstrated prognostic value, with defined cut‐off values enabling meaningful risk stratification and independently predicting mortality with acceptable performance [11, 12, 14]. However, paediatric candidemia differs substantially from adult disease in terms of clinical outcomes, epidemiological patterns, species distribution, underlying risk factors, and clinical presentation [15, 16]. These differences likely attenuate the relative contribution of individual score components and reduce the overall performance of standardised, guideline‐based scoring systems in predicting outcomes in children.

In addition, certain components of the score may be difficult to implement in routine paediatric practice. First, the recommended blood culture volumes required by the scoring system [e.g., up to 20 mL depending on body weight] may be challenging to obtain in younger children, particularly in critically ill or low‐weight patients. In routine clinical practice, however, inadequate blood culture volumes are frequently observed, with studies showing that fewer than half of paediatric blood cultures meet recommended volume criteria [17, 18]. Consistent with these observations, none of the patients in our cohort met the recommended blood culture volume criteria, highlighting a substantial gap between guideline recommendations and real‐world practice. Similarly, adherence to follow‐up investigations such as repeated blood cultures, ophthalmologic examinations, and echocardiography may be influenced by clinical stability, feasibility, and resource considerations rather than true deviations from quality care. These findings suggest that certain score components may reflect practical constraints rather than true deficiencies in care, thereby limiting the applicability and interpretability of the scoring system in real‐world paediatric settings.

Duyu et al. [10], demonstrated that the Paed‐EQUAL Candida Score has significant prognostic value in paediatric candidemia, with higher scores observed in survivors (median 18 vs. 15, *p* = 0.032) and each incremental point associated with improved survival (OR 0.703, *p* = 0.004). While these findings support the potential utility of the score in certain paediatric settings, the overall discriminative performance in our cohort was more modest, suggesting variability in predictive performance across different paediatric settings. Notably, while Duyu et al. [10], reported that individual components of the score did not independently predict mortality, our findings indicate that several key interventions, particularly central venous catheter removal, follow‐up blood cultures, and continuation of antifungal therapy for at least 14 days, were significantly associated with improved outcomes, including in multivariable analyses. In addition, interpretation of the catheter removal component should take catheter type into account, as ports were more common among non‐survivors and were removed less frequently than temporary or tunnelled central venous catheters in our cohort. This discrepancy may reflect differences in patient populations, clinical practices, and the relative contribution of specific management steps to outcomes. Importantly, our results suggest that while the composite score captures overall adherence to guideline‐based care, selected high‐impact components may play a more direct and clinically meaningful role in determining outcomes in real‐world paediatric practice.

This study has several limitations. First, its retrospective and single‐centre design may limit the generalisability of the findings. In addition, the retrospective nature of data collection may have introduced information bias, particularly in the assessment of certain score components based on clinical documentation. Second, the relatively small number of mortality events may have reduced the statistical power to detect associations for some variables. Furthermore, despite multivariable adjustment, residual confounding cannot be excluded. Additionally, detailed information regarding central venous catheter type was not systematically recorded and could not be reliably retrieved from the hospital information system because of the retrospective nature of the study. Therefore, we were unable to evaluate whether catheter removal practices differed according to catheter type, particularly for totally implantable venous access devices, which may be more difficult to remove than other central venous catheters.

In addition, certain components of the Paed‐EQUAL score could not be uniformly assessed in all patients due to the inherent challenges of paediatric clinical practice, particularly regarding recommended blood culture volumes. Finally, the score was applied retrospectively, which may not fully reflect real‐time clinical decision‐making and could have influenced its observed performance.

## 5 Conclusion

In conclusion, the Paed‐EQUAL Candida Score demonstrated limited ability to predict short‐term mortality and poor performance for long‐term outcomes in paediatric candidemia. Although higher adherence to guideline‐recommended care was associated with improved survival, the overall discriminative capacity of the score remained modest, largely due to its low specificity. These findings indicate that the Paed‐EQUAL Candida Score may be more appropriately used as a measure of quality of care rather than a prognostic tool in clinical decision‐making. Future studies should focus on developing paediatric‐specific models that integrate both adherence metrics and markers of disease severity to better capture the complex clinical and biological determinants of outcomes in paediatric candidemia.

## Author Contributions

**Turgut Ozdemir:** data curation, validation. **Osman Oguz Demir:** software, formal analysis, investigation. **Aylin Irmak Kuruc:** validation, formal analysis, investigation, data curation, methodology. **Ilgın Sirin:** data curation, validation. **Ali Bulent Cengiz:** writing – review and editing, supervision. **Hanife Avcı:** formal analysis, data curation, visualization. **Yasemin Ozsurekci:** project administration, writing – review and editing, supervision, conceptualization, investigation. **Kubra Aykac:** conceptualization, investigation, methodology, software, data curation, writing – original draft.

## Funding

This research received no external funding.

## Conflicts of Interest

The authors declare no conflicts of interest.

## Data Availability

The data that support the findings of this study are available on request from the corresponding author. The data are not publicly available due to privacy or ethical restrictions.
